# Supplementary figures and images for: Assessing the Potential Role of Cats (Felis catus) as Generators of Relevant SARS-CoV-2 Lineages during the Pandemic
Source: Pathogens. 2023 Nov 16;12(11):1361. doi: 10.3390/pathogens12111361 (PMC10675002; doi:10.3390/pathogens12111361)

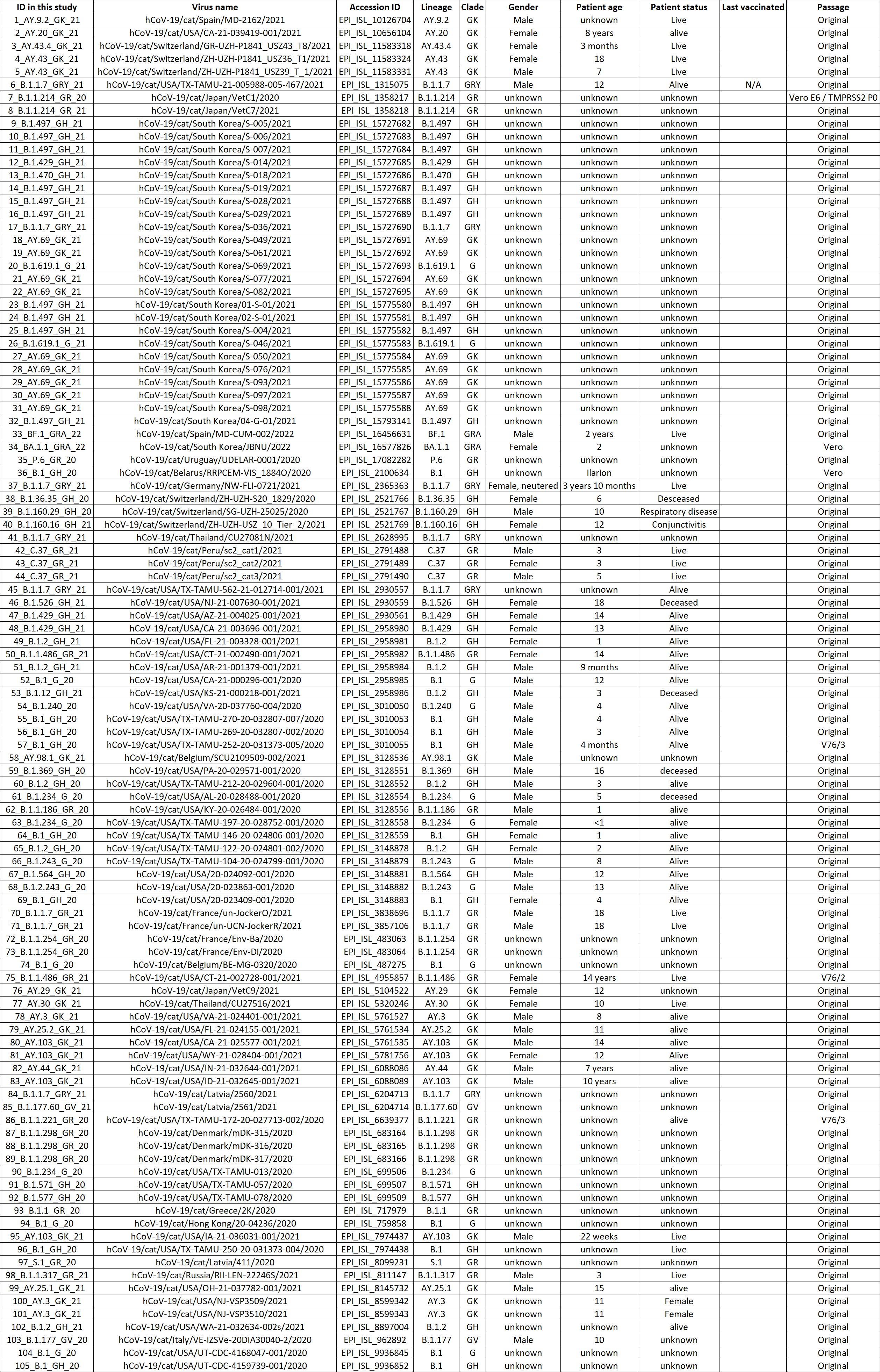

Supplement: Supplementary file 1 [file pathogens-12-01361-s001.zip › Supplementary file 1..tif]

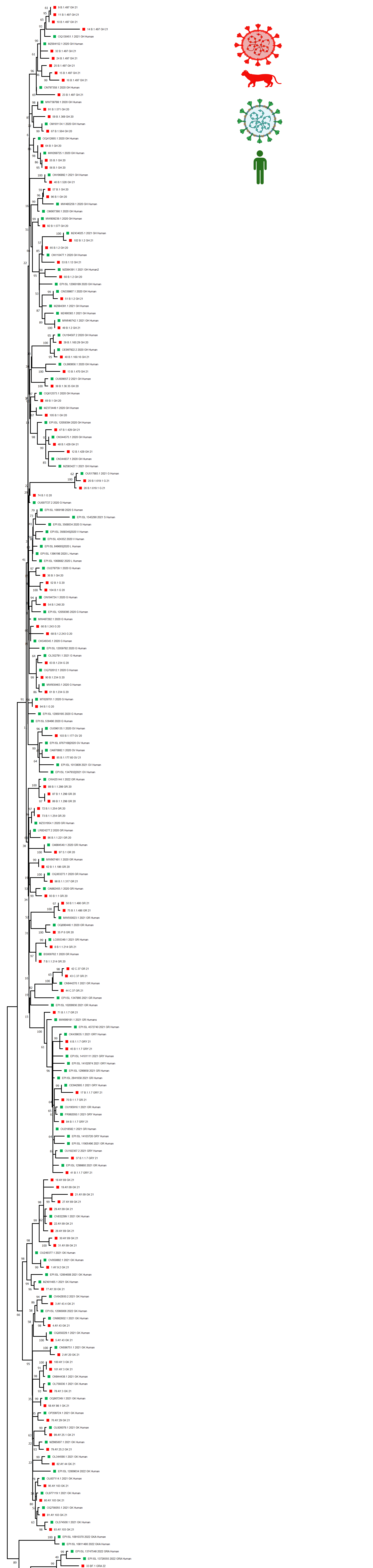

Supplement: Supplementary file 1 [file pathogens-12-01361-s001.zip › Supplementary file 3.tif]

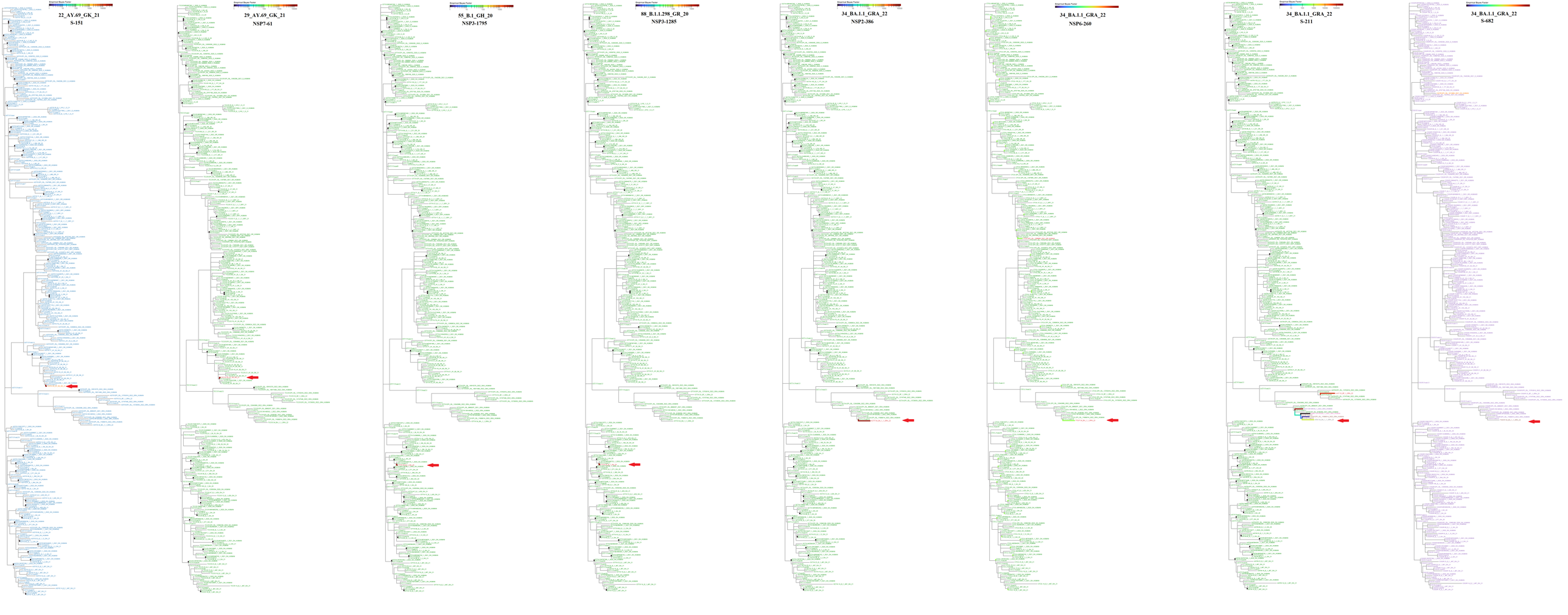

Supplement: Supplementary file 1 [file pathogens-12-01361-s001.zip › Supplementary file 4.tif]
